# The Relationship Between Serum Concentration of Interleukin-35 and FoxP3 Polymorphism in Patients Undergoing Coronary Artery Bypass Graft Surgery

**DOI:** 10.21470/1678-9741-2019-0377

**Published:** 2020

**Authors:** Farzaneh Dasturian, Nadereh Naderi, Gholamreza Farshidfar, Hossein Montazerghaem, Mahmood Khayatian, Sara Aghakhani Chegeni, Mahsa Rahimzadeh

**Affiliations:** 1 Molecular Medicine Research Center, Hormozgan Health Institute, Hormozgan University of Medical Sciences, Bandar Abbas, Iran.; 2 Department of Biochemistry, Faculty of Medicine, Hormozgan University of Medical Sciences, Bandar Abbas, Iran.; 3 Department of Immunology, Faculty of Medicine, Hormozgan University of Medical Sciences, Bandar Abbas, Iran.; 4 Cardiovascular Research Center, Hormozgan University of Medical Sciences, Bandar Abbas, Iran.

**Keywords:** Cardiopulmonary Bypass, Polymorphism Single Nucleotide, Body Mass Index, Arterial Pressure, Interleukins, Coronary Artery Bypass, Genotype, Polymerase Chain Reaction, Anti-Inflammatory Agents

## Abstract

**Objective:**

To investigate the association between interleukin-35 (IL-35) levels and single nucleotide polymorphisms (rs3761548, rs3761547) of the FoxP3 gene in coronary artery bypass grafting (CABG) patients.

**Methods:**

We conducted a prospective study including 140 patients, who were scheduled for elective isolated on-pump CABG with cardiopulmonary bypass (CPB) from January 2017 to September 2018 in the Jorjani heart center. Blood samples were collected before and 12 hours after the operation. Serum levels of IL-35 were measured by enzyme-linked immunosorbent assay and the pattern of genetic variations was assessed using single specific primer-polymerase chain reaction.

**Results:**

The serum concentrations of IL-35 after surgery were significantly higher than pre-surgery levels (18.4±8.3 *vs*. 9.89±3.2, respectively, *P*=0.002). There was no significant association between genotype frequencies of rs3761548 and rs3761547 and elevated IL-35 levels (*P*>0.05). There were significant associations between IL-35 levels and preoperative variables, including age (r=-0.34, *P*=0.047) and body mass index (r=-0.41, *P*=0.045), and intraoperative variables, including CPB time (r=0.4, *P*=0.02) and mean arterial pressure (r=-0.38, *P*=0.046), in carriers of the rs3761548 AA genotype.

**Conclusion:**

Serum IL-35 concentrations were significantly increased in CPB patients, which may contribute to the post-CPB compensatory anti-inflammatory response syndrome. IL-35 increased levels were not influenced by FoxP3 promoter polymorphisms (rs3761548, rs3761547).

**Table:** 

Abbreviations, acronyms & symbols			
AMI	= Acute myocardial infarction	HWE	= Hardy-Weinberg equilibrium
ARMS	= Amplification-refractory mutation system	ICU	= Intensive care unit
BMI	= Body mass index	IFN	= Interferon
CABG	= Coronary artery bypass grafting	IL	= Interleukin
CARS	= Compensatory anti-inflammatory response syndrome	IL-1ra	= Interleukin-1 receptor antagonist
COPD	= Chronic obstructive pulmonary disease	MAF	= Minor allele frequency
CPB	= Cardiopulmonary bypass	MAP	= Mean arterial pressure
CUF	= Conventional ultrafiltration	PCR	= Polymerase chain reaction
DNA	= Deoxyribonucleic acid	SIRS	= Systemic inflammatory response syndrome
EBI3	= Epstein-barr virus-induced gene-3	SNP	= Single nucleotide polymorphisms
EF	= Ejection fraction	TGF-β	= Transforming growth factor beta
ELISA	= Enzyme-linked immunosorbent assay	TNF	= Tumor necrosis factor
GAPDH	= Glyceraldehyde 3-phosphate dehydrogenase	Tregs	= Regulatory T cells
Hb	= Hemoglobin		

## INTRODUCTION

Cardiac surgery with cardiopulmonary bypass (CPB) leads to a well-recognized systemic inflammatory response syndrome (SIRS) and an accompanied compensatory anti-inflammatory response syndrome (CARS). The concept of CARS was proposed in 1997^[1]^ and defined as an adaptive reprogramming of the immune status trying to regulate acute pro-inflammatory response^[2]^. The compensatory phase apparently plays a notable role in generalized postoperative immunosuppression^[3]^ and the development of infectious complications after CPB^[1,4]^. Regulatory T cells (Tregs) are a specialized subpopulation of T cells which play a crucial role in maintaining immune homeostasis and control acute and chronic inflammation and are specified by forkhead/winged-helix family of transcription factors, called FoxP3^[5]^. Recent studies have addressed the functionality of FoxP3 Tregs during cardiac surgery and post-surgery complications. Several studies showed that serum Treg cytokines levels have significantly increased following CPB surgery^[6]^. These cells exert their suppressor activity through various molecular mechanisms, including cell contact-dependent mechanisms, production of transforming growth factor beta (TGF-β), interleukin-10 (IL-10), and IL-35 inhibitory cytokines^[7]^.

IL-35 is a Treg-dependent inhibitory cytokine, which is encoded by two separate genes called IL-12A and Epstein-barr virus-induced gene-3 (EBI3)^[8]^. IL-35 is required for maximal regulatory function of Tregs^[8]^ and it has an important part in regulating disease severity in autoimmune diabetes^[9]^, atherosclerosis^[10]^, unstable angina pectoris, and acute myocardial infarction^[11,12]^. The EBI subunit of this cytokine is a downstream target of FoxP3, a transcription factor that is required for Tregs development and function^[8]^.

Due to the probable roles that Tregs have in postoperative immunosuppression, the importance of post-CPB infectious complications, and the lack of published paper concerning IL-35 and its related regulatory gene, FoxP3, in CPB patients, we carried out a prospective study on Iranian patients undergoing coronary artery bypass grafting (CABG). Since FoxP3 plays a major role in expression of the EBI subunit of IL-35, we measured the plasma IL-35 levels and investigated whether these levels are modulated by FoxP3 polymorphisms (rs3761548, rs3761547).

## METHODS

### Study Design

In this prospective before-and-after study, 140 patients, aged between 25 to 80 years, scheduled for elective isolated on-pump CABG between January 2017 and September 2018 in the Jorjani heart center, Bandar Abbas, Iran, were included. All procedures performed in studies involving human participants were in accordance with the ethical standards of the institutional and/or national research committee and with the 1964 Helsinki declaration and its later amendments or comparable ethical standards. All subjects signed consent forms. The ethical committee of the Hormozgan University of Medical Sciences confirmed the study (committee’s reference number: HUMS.REC.1395.81). Anesthesia and surgical method, surgeons and physicians, and medication protocol were the same for all patients. The exclusion criteria were as follows: patients younger than 25 or older than 80 years, occurrence of an acute myocardial infarction within the last two months, an ejection fraction (EF) below 30%, all kinds of emergency surgery, liver and kidney diseases, hepatic failure, neurological diseases (psychosis or dementia), current inflammatory or infectious condition, addiction, presence of a systemic inflammatory disease or receiving immunosuppressive drug, any type of metastatic disease and history of chronic obstructive pulmonary disease (COPD), and those who had undergone emergency surgery or who had died during or seven days after surgery. Patients with diagnosed coagulopathy or low platelet count, reoperation, postoperative complications that resulted in prolonged hospitalization in the intensive care unit (ICU) (longer than four days), emergency surgery, combined surgical procedures and acute coronary syndrome, and those with any type of transfusion three months prior to surgery were also excluded. Postoperative variables such as duration of ICU stay and length of hospital stay were recorded.

### Pulsatile Cardiopulmonary Bypass Technique

For the standard preanesthetic medication, 1 mg alprazolam was administered orally eight hours before surgery; no medication was used for hemodynamically unstable patients. All patients received balanced anesthesia as the following: denitrogenation was performed with 100% oxygen; then anesthetic induction was performed with midazolam (0.05 mg/g), sufentanil (0.5 µg/kg), or fentanyl (5 µg/kg), etomidate (2 mg/kg), and cisatracurium (0.2 mg/kg) as a neuromuscular blocker. After intubation, all patients were ventilated with a fraction of inspired oxygen of 60%, volume-controlled and pressure-limited ventilation (25 mmHg), tidal volume ranging from 6 to 8 mL/kg, respiratory rate of 12 breaths/min, gas flow of 1.0 L/min and positive end-expiratory pressure of 5 mmHg. Anesthesia was maintained with a propofol infusion (100 µg/kg) and intermittent doses of sufentanil or fentanyl and cisatracurium. A new aliquot of midazolam and a neuromuscular blocker were administered before surgery. Intraoperative hydration was performed; in patients without hemodynamic instability, the red blood cell transfusion threshold was established as hemoglobin (Hb) < 7 mg/dL, and in those with hemodynamic instability, it was established as Hb < 9 mg/dL.

Heparin with concentration of 300-400 U/kg was used as anticoagulant to maintain an activated clotting time greater than 450 seconds. The aorta and the right atrium were cannulated at the first step of CPB. A pulsatile roller pump (Stockert Instrumente GmbH, Germany) and an oxygenator (Dideco Compact Flo Evo, Sorin Group, Milan, Italy) were used. Lactated Ringer’s solution (1000 mL) and colloid solution (Voluven, 500 mL) were used as the pump prime solution to maintain a hematocrit level of 26±2. The pump flow was 2.2-2.4 L/m^2^/min and the pulsatile arterial pressure was maintained between 50-70 mmHg. The patient’s body temperature was cooled down to 30°C. Cross-clamp time and CPB time (minutes) were recorded. Potassium/blood cardioplegic solution was administered every 20 minutes and Isolyte S solution (4°C) was applied to the surface of the heart at the same intervals. Other strategies for better outcome were as following: conventional ultrafiltration (CUF) (30-50 mL/kg) during CPB, pulsatile perfusion flow after cardiac arrest until beginning of heart rate after aorta declamping, administration of adenosine at every cardioplegia delivery (peri-conditioning), administration of vitamin C (2 g before aorta declamping), and priming solution composed of other additive agents, like laxis, sodium bicarbonate, tranexamic acid, and heparin sodium.

### Blood and Serum Collection and IL-35 Analyses

A predesigned questionnaire was used to document the demographic and clinical data. Blood samples were collected before operation and 12 hours postoperatively. Centrifugation was performed at 2000 g for 15 min at 4 °C (sigma 2-16KL), the serum was obtained and stored at -75 °C. IL-35 was measured using enzyme-linked immunosorbent assay (ELISA) (Zell bio, Germany). Standard curve was developed and used for the calculation of IL-35 concentration. Minimum detectable dose (lower limit of detection) of IL-35 was < 0.27 ng/mL.

### FoxP3 Single Nucleotide Polymorphisms Selection and Genotyping Analyses

FoxP3 single nucleotide polymorphisms (SNPs) were selected according to the previously published literature. Those SNPs which were associated with the disease and located in the promoter or intron sequences with the minor allele frequency (MAF) of ≥ 5% were considered. Finally, two SNPs, rs3761548 and rs3761547, were selected and analyzed in the study subjects. Genomic deoxyribonucleic acid (DNA) was extracted from 5 mL peripheral venous blood (Diatom DNA Extraction kit, Thermo Fisher Scientific, United States of America). Amplification-refractory mutation system (ARMS) assay was used to genotype the selected SNPs. Primers were designed using Primer3 software and NCBI Primer Blast tool (Table 1). Glyceraldehyde 3-phosphate dehydrogenase specific primers were used as the internal positive control. The following reactions were used for the ARMS: 0.2 µl of prepared DNA, 0.6 µl of each primer pair (stock concentration of 20 pmol/µL), 0.2 µl of *Taq* DNA polymerase buffer (5 unit/µl), 0.2 µl of deoxyribose nucleoside triphosphate (stock concentration of 20 mM), 2 µl of polymerase chain reaction buffer 10x, 0.2 µl of MgCl_2_ (stock concentration of 50 mM), and sterile double distilled water to a final volume of 20 µl. The amplification program was as follows: initial denaturation at 95 °C for five minutes, 30 cycles of amplification with denaturation at 94°C for 30 s, primer annealing at 60 °C for rs3761548 and rs3761547, and 58 °C and 62 °C for rs2232365 and rs4824747, respectively, for 45 s, extension at 72 °C for 60 s, and final extension step of seven minutes at 72 °C. Amplified fragments were electrophoresed on 2% agarose (Sigma Chemical Co.) gel, stained with Gel red dye (Sigma Chemical Co.), and photographed.

**Table 1:** Primer sequences used for single specific primer-PCR.

SNP	Primer sequences	Size of PCR product
rs3761548	F1 5'-CTGGCTCTCTCCCCAACTGA-3'	332 bp
	F2 5'-TGGCTCTCTCCCCAACTGC-3'	
	R-ACAGAGCCCATCATCAGACTCTCTA-3'	
rs3761547	F5'-GCTTTCTATTCTGTTCTCTTCCC-3'	416 bp
	R1 5'-TGCAGGGCTTCAAGTTGACAAC-3'	
	R2 5'-TGCAGGGCTTCAAGTTGACAAT-3'	
GAPDH	F5'-GCAGCCCTGGAGCCTTCA-3'	581 bp
	R5'-TTACCATATACCCAAGGGAGCC-3'	

GAPDH=glyceraldehyde 3-phosphate dehydrogenase; PCR=polymerase chain reaction; SNP=single nucleotide polymorphisms

### Statistical Analysis

Pearson’s chi-square test was used to analyze deviation from Hardy-Weinberg equilibrium (HWE). We used the Open Source Epidemiologic Statistics for Public Health software, version 3.01, for the estimation of sample size and power of study. Chi-square test was used for comparing categorical variables. The association between IL-35 and demographic data was analyzed using multivariable logistic regression and adjusted for other covariates. *P*<0.05 was considered statistically significant. Statistical analysis was performed using the IBM SPSS Statistics software, version 20.0 (SPSS Inc., Chicago, Illinois, United States of America).

## RESULTS

### Patients’ Characteristics

As it was shown in Table 2, preoperative variables were recorded during the patients’ admission to the hospital. The mean age of the 140 study subjects was 57.9±10.3 years and 78 males and 62 females were included. The mean body mass index (BMI) of the patients was 24.1±4.3. During and after surgery, the patients’ intra/postoperative data were collected (Table 3). DNA extraction was successfully performed from all samples and ARMS success rate was sufficient for analyses of the genotypes. Patients’ demographics and pre/intra/postoperative variables were analyzed across two SNPs, rs3761548 and rs3761547. No significant differences were observed (Table 4).

**Table 2:** Demographic and preoperative clinical data of 140 patients undergoing on-pump coronary artery bypass grafting.

Patients' characteristics (n=140)	
Age (years)	57.9±10.3
Sex (%)	
Male (n=78)	55.7
Female (n=62)	44.3
BMI (kg/m^2^)	24.1±4.3
Baseline plasma creatinine (mg/dL)	1±0.35
Systolic blood pressure (mmHg)	126.9±31.1
Diastolic blood pressure (mmHg)	74.4±15.2
Diabetes (%)	51%
Hypertension (%)	43.1%
Current smoker (%)	56%
Dyslipidemia (%)	66%
Previous AMI (more than 2 months ago) (%)	33%
Previous angioplasty (%)	31%
Ejection fraction (%)	49.6±10.7

AMI=acute myocardial infarction; BMI=body mass index

**Table 3:** Intraoperative and postoperative variables in patients undergoing on-pump coronary artery bypass grafting (n=140).

Variables	
Cross-clamp time (min)	81±31.5
CPB time (min)	129.3 ±35.9
One graft (%)	22%
Two grafts (%)	33%
More than 2 grafts (%)	45%
Left main stenosis (%)	23%
Arterial flow (mL/kg/min)	3823.3±521.2
MAP during CPB (mmHg)	66.5±3.7
ICU stay (days)	2±1.1
Length of hospital stay (days)	8±2.3

Data presented as mean±standard deviation or percent CPB=cardiopulmonary bypass; ICU=intensive care unit; MAP=mean arterial pressure

**Table 4:** Analyses of patients' demographics and pre/intra/postoperative variables across two single nucleotide polymorphisms, rs3761548 and rs3761547.

Variable	rs3761548				rs3761547			
	AA	AC	CC	*P*-value	CC	CT	TT	*P*-value
Age (years)	57.5±8.3	57.6±11.8	58.1±10.2	0.9	54±7.8	57.8±10.3	55.6±6.3	0.4
BMI (kg/m^2^)	24.5±4.1	24.7±5	24±3.6	0.8	23.9±3.1	24.4±4.2	24.7±3.7	0.4
Baseline plasma creatinine (mg/dL)	1.07±0.4	0.9±0.2	1.08±0.4	0.05	0.9±0.2	1.03±0.4	1.1±0.2	0.5
Systolic blood pressure (mmHg)	126.4±61.1	129.8±22.6	126.2±26.7	0.9	121.5±30.3	127±32.1	129.4±27.6	0.5
Diastolic blood pressure (mmHg)	81.6±18.2	71.2±17.9	74.5±12]	0.3	80.4±24.1	73.9±15.5	75.6±17.5	0.4
Diabetes (%)	34.5	42.5	38.1	0.2	50	41.5	43.2	0.5
Hypertension (%)	58.6	58.5	57.1	0.9	66.1	57	59.4	0.5
Current smoker (%)	36.8	19.2	26.5	0.4	50	25.3	26.6	0.3
Dyslipidemia (%)	58.2	66.6	53.1	0.4	62.3	68.4	55	0.5
Previous AMI (more than 2 months ago) (%)	28.5	33.5	24.2	0.6	36.3	25.7	30.1	0.7
Previous angioplasty (%)	28.3	33.2	31.3	0.6	32.2	31.6	29.3	0.8
Ejection fraction (%)	49.6±11.2	45.3±10.5	47.8±7.6	0.2	47.7±10.2	46.6±9.9	49.2±11.5	0.7
Cross-clamp time (min)	79.7±27.5	82.4±40.2	81.3±24.5	0.9	80.7±14.8	81.9±31.9	78.7±22.3	0.7
CPB time (min)	131±32.8	129.6±46.6	128.5±26.9	0.9	115.2±31.5	130.3±36.3	127.4±30.7	0.5
One graft (%)	26.3	25.7	19.3	0.3	22	24.7	21.1	0.4
Two grafts (%)	35.1	34.3	36.5	0.9	33.7	30.2	36.1	0.5
More than 2 grafts (%)	38.6	40	42.2	0.8	44.3	45.1	42.8	0.8
Left main stenosis (%)	22.7	20.7	24.2	0.8	21.2	24.5	22.3	0.6
Arterial flow (mL/kg/min)	3603.3±821.2	3828±566.2	3924±652.6	0.6	3703.3±821.2	3954±621.7	3783±757.5	0.3
MAP during CPB (mmHg)	61.5±3.1	58.9±2.6	66.3±4.1	0.4	67.7±6.5	65.3±3.5	66.3±4.1	0.8
ICU stay (days)	2.2±1.5	2.7±1.4	2.1±1.1	0.9	2.1±1.3	2.3±1.1	2±1.4	0.8
Length of hospital stay (days)	9.4±3.3	8.1±1.7	7.6±2	0.8	8±3.4	7.8±1.5	8.2±1.7	0.8

AMI=acute myocardial infarction; BMI=body mass index; CPB=cardiopulmonary bypass; ICU=intensive care unit; MAP=mean arterial pressure

### Comparison of IL-35 Serum Level Before and After Operation

**Fig. 1:**
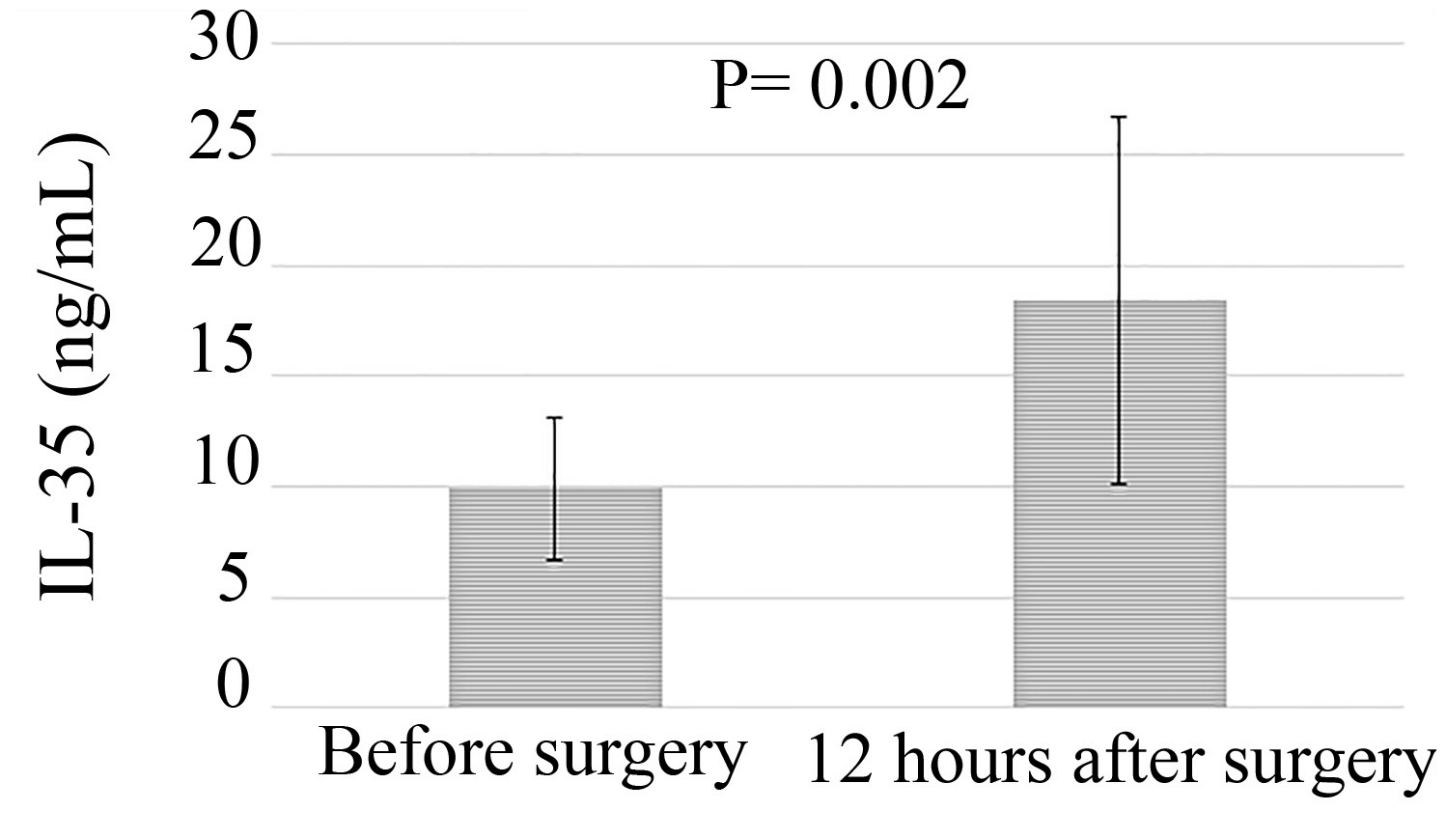
Comparison of interleukin-35 (IL-35) serum levels in patients undergoing cardiopulmonary bypass before and 12 hours after operation (n=140).

IL-35 serum concentrations were examined in patients (n=140) before and 12 hours after operation (Figure 1). Results showed that IL-35 serum level was significantly higher 12 hours after the surgery than before it (18.4±8.3 *vs*. 9.89±3.2, respectively, *P*=0.002).

### Association between Serum IL-35 and FoxP3 Polymorphism

**Fig. 2:**
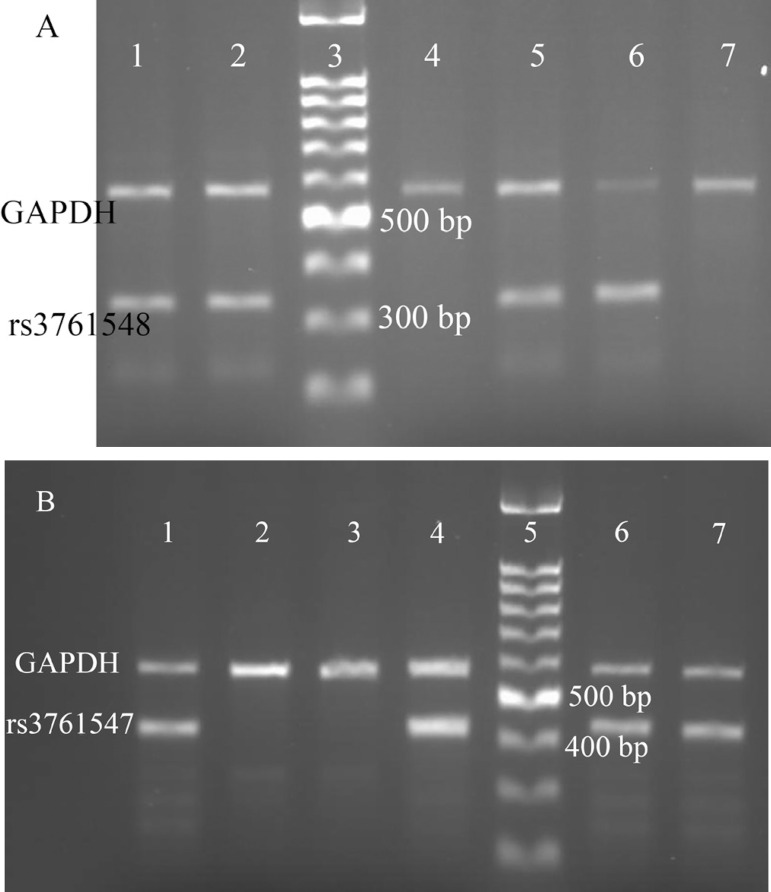
Gel electrophoreses of the polymerase chain reaction products for analysis of FoxP3 polymorphisms. Glyceraldehyde 3-phosphate dehydrogenase (GAPDH) was used as internal positive control. A) FoxP3 rs3761548 polymorphisms; lane 1,2: AC heterozygote; lane 3: 100 bp molecular weight marker; lane 4,5: AA homozygote; lane 6,7: CC homozygote. B) FoxP3 rs3761547 polymorphisms; lane 1,2: TT: homozygote; lane 3,4: CC homozygote; lane 5: 100 bp molecular weight marker, lane 6,7: CT: heterozygote.

As it was shown in Figures 2A and B, for each SNP, three genotypes were seen by the ARMS method (for rs3761548: CC, AC, AA; for rs3761547: TT, CT, CC). The frequency of each genotype and the deviation from HWE were shown in Table 5. The genotype frequencies of rs3761548 were CC (25.7%), AC (43.6%), and AA (30.7%). And the frequencies of rs3761547 genotype were TT (28.6%), CT (52.8%), and CC (18.6%). As it was shown in Table 6, the association between rs3761548 and rs3761547 polymorphisms and serum IL-35 levels was examined. IL-35 serum concentration was compared between different genotypes of SNPs. No significant associations were observed between IL-35 serum levels and SNPs genotypes before and 12 hours after operation (*P*˃0.05).

**Table 5:** FoxP3 single nucleotide polymorphisms genotype frequencies in 140 patients undergoing on-pump coronary artery bypass grafting.

	Frequency (number/%)		Hardy-Weinberg equilibrium
rs3761548	AA	43/30.7	0.1
	AC	61/43.6	
	CC	36/25.7	
rs3761547	CC	26/18.6	0.4
	CT	74/52.8	
	TT	40/28.6	

**Table 6:** Comparison of interleukin-35 (IL-35) serum concentration before and 12 hours after operation in 140 patients based on SNP genotypes.

SNP rs3761548	IL-35 serum concentration (ng/mL) before surgery	IL-35 serum concentration (ng/mL) 12 hours after surgery
**Genotypes**		
CC	9.5±5.4	20. 7±12.1
AC	6.3±2.5	18.7±10.1
AA	6.3±3.7	17.2±11.8
*P*-value	0.1	0.6
SNP rs3761547		
**Genotypes**		
TT	4.5±2.2	8.1±7.4
CT	7.7±5.6	19.4±14.3
CC	6.7±4.2	14.4±10.5
*P*-value	0.9	0.9

SNP=single nucleotide polymorphisms

### Association between IL-35 Serum Level and Pre/Intra/Postoperative Variables

The correlation between IL-35 serum levels and pre/intraoperative variables before and post operation and pre/intra/postoperative variables were analyzed. No significant differences were observed. In the next step, patients were categorized according to the different genotypes (three genotypes for each SNP). Analyses revealed that in those patients carrying SNP rs3761548 AA genotype, there was negative correlation between age and IL-35 serum level before operation (Table 7). No significant correlation was observed between age and IL-35 serum level 12 hours postoperatively. IL-35 serum level 12 hours after operation was negatively correlated to BMI and mean arterial pressure (MAP) during CPB (correlation coefficient (r)=-0.41 and -0.38, *P*=0.045 and 0.046, respectively). There wasn’t any correlation between the serum levels of IL-35 before operation and BMI (*P*>0.05). CPB time was positively correlated to serum IL-35 12 hours postoperatively (r=0.4, *P*=0.02). There was no significant correlation between pre/intra/postoperative variables and other studied genotypes.

**Table 7:** Correlation analysis of the interleukin-35 (IL-35) serum level and pre/intraoperative variables. All variables were analyzed according to rs3761547 and rs3761548 genotypes. Only significant correlations were shown.

Variables in patients carrying SNP rs3761548 AA genotype	Serum IL-35 before surgery		Serum IL-35 12 hours postoperatively	
	*R* value	*P*-value	*R* value	*P*-value
Age	-0.34	0.047	-	-
BMI	-	-	-0.41	0.045
CPB time	-	-	0.4	0.02
MAP during CPB	-	-	-0.38	0.046

BMI=body mass index; CPB=cardiopulmonary bypass; MAP=mean arterial pressure; SNP=single nucleotide polymorphisms

## DISCUSSION

The principal and novel findings of this study were as follows: (1) IL-35 levels were significantly increased after CPB; (2) there was no association between IL-35 levels and pre/intra/postoperative variables, but while categorizing CPB patients to the different FoxP3 SNPs (rs3761548 and rs3761547) genotypes, we found significant associations between preoperative (age, BMI) and intraoperative (CPB time and MAP) variables and IL-35 levels in carriers of the rs3761548 AA genotype; and (3) the FoxP3 promoter polymorphisms (rs3761548, rs3761547) have no impact on the elevated levels of IL-35 after cardiac surgery with CPB.

Previous studies showed that IL-35 is an anti-inflammatory cytokine, which limits early T cell rest on the G1 phase of cell division, blocks the proliferation of Th1 and Th17 cells^[13]^, represses GATA3 and IL-4 expression, limits Th2 proliferation^[14]^, and silence acute pro-inflammatory genes^[15]^.

Moreover, IL-35 promotes the regulatory activity of Tregs^[8,16]^ and induces the development of a newly identified population of CD4+ Tregs, iTr35^[17]^. Our result on the increased IL-35 production during CPB is consistent with earlier studies which showed that release of anti-inflammatory mediators are an unavoidable accompaniment of CPB^[18-20]^.

Previous studies support a post-CPB hyporesponsiveness of whole blood cells^[21]^ and a marked reduction in Th1-related cytokines, including IL-12, IL-2, interferon (IFN)-γ, and tumor necrosis factor (TNF)^[22]^. Sablotzki et al.^[22,23]^ studies showed that the phased anti-inflammatory cytokine response in CPB patients, commencing with IL-10 and TGF-b, was nearly three-fold higher than in patients with septic shock. McBride et al.^[20]^ showed significantly elevated levels of IL-10, 10 min after the aortic cross-clamp, which was followed by increases in interleukin-1 receptor antagonist (IL-1ra) and TNF soluble receptors, two and 24 hours and 24 and 48 hours post-CPB, respectively. Our findings are also consistent with Cheng et al. results about the postoperative elevated levels of IL-19, which like IL-35 inhibits T-cell activity, upregulates FoxP3 expression in CD4 T cells, and increases the differentiation of Tregs^[19,20]^.

It seems logical that IL-35 acts as a part of post-CPB anti-inflammatory phase and represents an attempt of the host to counteract the postoperative inflammatory responses, characterized by IL-1, IL-6, TNF^[24,25]^, and chemokines release, and of inflammatory cells, specially neutrophils activation^[24,26]^ and the subsequent microcirculation and organ dysfunction^[27]^. Regarding this, we did not find any association between rs3761548, rs3761547 FoxP3 promoter polymorphisms and the IL-35 serum levels increase after CPB, but our findings about the association between IL-35 levels and preoperative (age, BMI) and intraoperative (CPB time and MAP) variables in carriers of the rs3761548 AA genotype are somehow consistent with Bassagh et al.^[28]^ study in peptic ulcer patients, Jafarzadeh et al.^[29]^ study in multiple sclerosis, and Chatrabnous et al.^[30]^ study in prostate cancer patients which showed that the IL-35 levels were significantly different in subjects with AA genotype.

## CONCLUSION

In conclusion, there wasn’t any association between IL-35 serum levels increase after CPB and rs3761548, rs3761547 polymorphisms, but due to the IL-35 key role in inflammatory effector cells suppression^[31]^, one can assume that postoperative IL-35 elevation, along with IL-10, TGF, and IL-19, may act as an integral part of post-CPB CARS, trying to return the body to homeostasis and may play a role in development of post-CPB infectious complications. However, we were limited by a relatively small sample size and caution is still appropriate in generalizing our results. Additional studies with larger number of patients to evaluate the role of IL-35 and FoxP3 in post-CPB anti-inflammatory state and its potential impact on postoperative morbidity may be helpful in understanding and preventing the development of infectious complications in CPB patients.

**Table:** 

Authors' roles & responsibilities	
FD	Substantial contributions to the conception or design of the work; or the acquisition, analysis, or interpretation of data for the work; agreement to be accountable for all aspects of the work in ensuring that questions related to the accuracy or integrity of any part of the work are appropriately investigated and resolved
NN	Substantial contributions to the conception or design of the work; or the acquisition, analysis, or interpretation of data for the work
GF	Final approval of the version to be published
HM	Drafting the work or revising it critically for important intellectual content
MK	Final approval of the version to be published
SAC	Agreement to be accountable for all aspects of the work in ensuring that questions related to the accuracy or integrity of any part of the work are appropriately investigated and resolved
MR	Substantial contributions to the conception or design of the work; or the acquisition, analysis, or interpretation of data for the work
